# Cooling-Sensitive TRPM8 Is Thermostat of Skin Temperature against Cooling

**DOI:** 10.1371/journal.pone.0017504

**Published:** 2011-03-02

**Authors:** Koji Tajino, Hiroshi Hosokawa, Shingo Maegawa, Kiyoshi Matsumura, Ajay Dhaka, Shigeo Kobayashi

**Affiliations:** 1 Department of Intelligence Science and Technology, Graduate School of Informatics, Kyoto University, Kyoto, Japan; 2 Faculty of Information Science and Technology, Osaka Institute of Technology, Hirakata, Osaka, Japan; 3 Department of Biological Structure, University of Washington, Seattle, Washington, United States of America; Dalhousie University, Canada

## Abstract

We have shown that cutaneous cooling-sensitive receptors can work as thermostats of skin temperature against cooling. However, molecule of the thermostat is not known. Here, we studied whether cooling-sensitive TRPM8 channels act as thermostats. TRPM8 in HEK293 cells generated output (*y*) when temperature (*T*) was below threshold of 28.4°C. Output (*y*) is given by two equations: At *T* >28.4°C, *y* = 0; At *T* <28.4°C, *y*  =  -*k*(*T* – 28.4°C). These equations show that TRPM8 is directional comparator to elicits output (*y*) depending on negative value of thermal difference (Δ*T  =  T* – 28.4°C). If negative Δ*T*-dependent output of TRPM8 in the skin induces responses to warm the skin for minimizing Δ*T* recursively, TRPM8 acts as thermostats against cooling. With TRPM8-deficient mice, we explored whether TRPM8 induces responses to warm the skin against cooling. In behavioral regulation, when room temperature was 10°C, TRPM8 induced behavior to move to heated floor (35°C) for warming the sole skin. In autonomic regulation, TRPM8 induced activities of thermogenic brown adipose tissue (BAT) against cooling. When menthol was applied to the whole trunk skin at neutral room temperature (27°C), TRPM8 induced a rise in core temperature, which warmed the trunk skin slightly. In contrast, when room was cooled from 27 to 10°C, TRPM8 induced a small rise in core temperature, but skin temperature was severely reduced in both TRPM8-deficient and wild-type mice by a large heat leak to the surroundings. This shows that TRPM8-driven endothermic system is less effective for maintenance of skin temperature against cooling. In conclusion, we found that TRPM8 is molecule of thermostat of skin temperature against cooling.

## Introduction

In mammals, thermostat is a key device to maintain body temperature against changes in ambient temperatures. Many investigators have studied thermostats [1], [2], [3], [4], [5], [6]. Since Adrian [7], it has been assumed that thermo receptors are transducers (or sensors) that change temperature into the firing-rate code of impulse trains, travelling to the brain. Along this line, a target neuron receiving impulses from thermal receptors has been assumed to be “thermostat” that compares skin temperature with a set-point temperature at the level of the firing-rate code, and generates impulses as drive signal inducing thermoregulatory responses [1], [3], [4], [8].

If thermo receptors are transducers of temperature, their outputs should be related to temperature strictly. However, output of thermo receptors shows adaptation [7], [9], [10], [11], [12], even when temperature is constant. In addition, receptors show threshold response to temperature changes [10], [11]. Moreover, TRPM8, a member of thermo TRP channel family [13], responds to menthol and icilin as well as temperature [10], [14]. These results deny the assumption that receptors are transducers. Therefore, it is impossible for cerebral target neurons to detect skin temperature from afferent impulses sent from cooling-sensitive receptors. In fact, the “thermostat” neuron hypothesized in the brain [3], [4], [8], [15] has not been found. By lack of the “thermostat”, regulation of skin temperature is not explained.

In contrast, we have shown that cutaneous cooling-sensitive receptor itself can act as thermostat, comparing skin temperature with its threshold temperature [11], [16]. Single-channel recording shows that channel activities appear when temperature is lower than threshold temperature [11]. These results indicate that phase transition is a biological direct method for comparison, different from artificial indirect one using a code. If cooling-induced activities of receptors elicit responses to warm the skin recursively, a negative feedback loop is formed for regulation of skin temperature against cooling [16]. However, molecule of the thermostat has not been clarified.

Recently, cooling- and menthol-sensitive TRPM8 [10], [14] has been identified. TRPM8 is expressed in C and Aδ sensory fibers [17], [18] of cutaneous sensory nerves [19], [20]. Experiments of TRPM8-deficient mice show that TRPM8 is required for cold sensation [17], [21], [22]. Menthol application to the whole trunk skin [23] and intravenous menthol injection [24] induce behavioral responses to warm the skin. In contrast, TRPM8-induced autonomic thermoregulatory responses are not fully analyzed [23], [25], [26]. Here, with TRPM8-deficient mice, we studied whether TRPM8 in the skin induces behavioral and autonomic responses to warm skin against room cooling.

## Results

### TRPM8 is comparator, but not transducer

**Figure 1:**
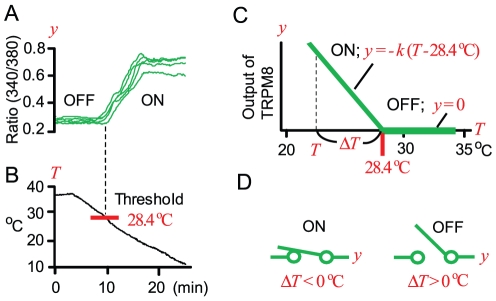
TRPM8 acting as comparator of temperature. (A, B) Ca^2+^ level (ratio) in five HEK293 cells expressing TRPM8. When temperature (*T*) was decreased slowly from 38 to 10°C (B), activities (output, *y*) of TRPM8 appeared at threshold temperature (28.4±0.5°C, mean ± SEM; n = 5) and increased with a fall in temperature. (C) Relationship between output (*y*) and temperature (*T*). Threshold response in (A) is shown approximately by two lines (green), which are expressed by two linear equations. When *T* is above 28.4°C, output (*y*) is 0. When *T* is below 28.4°C, output (*y*)  =  -*k*(*T* – 28.4°C). For explanation of the automatic control theory, thermal difference, Δ*T* ( =  *T* – 28.4°C), is defined. When *T* is higher than 28.4°C, Δ*T* is positive. When *T* is lower than 28.4°C, Δ*T* is negative. Thus, *y* increases with expansion of negative Δ*T*. (D) Representation of TRPM8 by a cooling-sensitive switch. When Δ*T* is positive, this switch is turned off. When Δ*T* is negative, this switch is turned on and *y* increases with expansion of negative Δ*T* as shown in C.

We examined thermal responses of TRPM8 channels expressed in HEK293 cells by calcium imaging [12]. When temperature (*T*) was decreased slowly from 38 to 10°C (Fig. 1B), activities (*y*) of TRPM8 appeared at threshold temperature (mean, 28.4°C), and increased with a fall in temperature, consistent with prior studies [10], [14], [17]. To explore a role of TRPM8, we schematically showed relationship between output (*y*) and temperature (*T*) in Fig. 1C. Due to the threshold response, output (*y*) is expressed by two equations:

Equation 1: 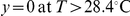


Equation 2: 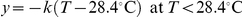


Threshold temperature (28.4°C) is constant specific to TRPM8, and *T* is variable applied to TRPM8. In Equation 2, -*k* (<0) is a slope constant. Two equations show that TRPM8 has silent (Equation 1) and active (Equation 2) phases, and that phase transition occurs at 28.4°C [11]. In terms of control theory, TRPM8 functions as directional comparator judging whether *T* is lower than 28.4°C, and generates output (*y*) proportionately with a negative thermal difference (Δ*T*  =  *T* – 28.4°C). For instance, when *T* is 22.4°C, TRPM8 enters active phase (Equation 2), and generates output (*y*) according to −6°C.

To aid comprehension, we symbolically show TRPM8 by a cooling-sensitive switch (Fig. 1D). At Δ*T* >0°C, this switch is turned off. At Δ*T* <0°C, this switch is turned on and output (*y*) increases with a fall in *T* from 28.4°C (Fig. 1C). In sensory neurons, output of sensory receptors generates receptor potential [11], eliciting impulse trains on sensory nerves [17]. If negative Δ*T*-dependent impulse activities induce thermoregulatory responses to warm the skin for minimizing Δ*T* recursively, TRPM8 switch acts as thermostats of skin temperature having 28.4°C as set-point temperature against cooling [16]. Then, intensity of the thermoregulatory activities (e.g., cold sensation and BAT activities) increases proportionately with expansion of Δ*T*, which corresponds to the proportional control in technology [16].

TRPM8 is also activated by menthol, when menthol concentration is higher than threshold level of 20 µM [10]. This shows that TRPM8 acts as directional comparator of menthol concentration, generating output (*y*) depending on the positive difference from 20 µM to menthol concentration. In addition, TRPM8 is activated by icilin, when its concentration is higher than 0.1 µM [10]. These properties indicate that TRPM8 are polymodal comparator of several kinds of stimuli. In later experiments, menthol is applied to the skin to explore TRPM8-driven thermogenic activities at neutral temperature [23]. It should be notified, however, that even when menthol-induced activities of TRPM8 elicits responses to warm the skin, negative feedback loops are not formed, because responses to warm the skin do not reduce menthol concentration in the skin.

In contrast, if TRPM8 is transducer of temperature, output (*y*) of TRPM8 should be expressed by one continuous function of *T* [8], In fact, in the transducer hypothesis [7], threshold responses of sensory receptors were not described at all. However, threshold response (Fig. 1) is essential feature of TRPM8 [10], [14], [17], and output (*y*) is not expressed by one function of *T*. Moreover, output of TRPM8 is changed by menthol, icilin and other chemicals, even when *T* is constant [10], [14], [27]. When menthol and *T* changes are applied in turn, output of TRPM8 shows complicated trace, different from *T* [14]. Thus, target neurons can hardly detect skin temperature from impulses sent from TRPM8. These results reject the hypothesis that the “thermostat” of skin temperature is present in the brain [1], [4], [8].

### TRPM8-driven behavior for warming the sole skin against room cooling

**Figure 2:**
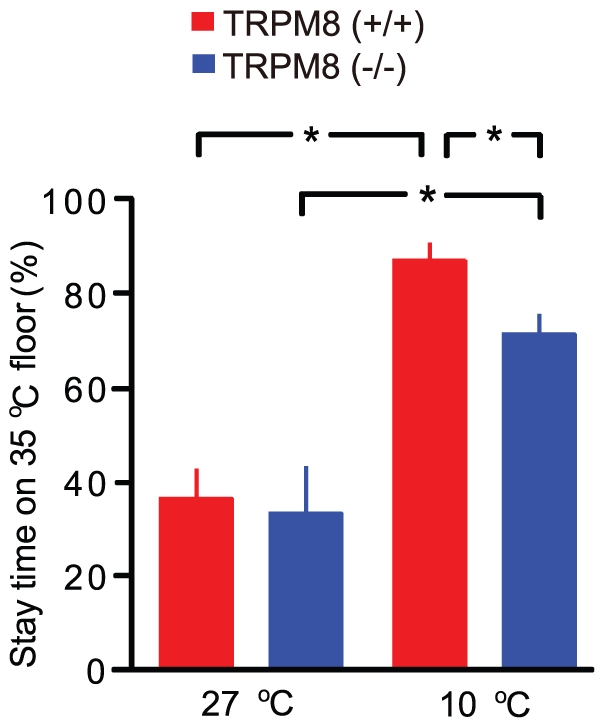
TRPM8-driven heat-seeking behavior against room cooling. When room temperature was neutral (27°C), stay time on 35°C floor was not different between wild-type and TRPM8-deficient mice. At 10°C, stay time on 35°C floor was increased in both mice. However, stay time on 35°C floor in wild-type mice was longer than that in TRPM8-deficient mice (n = 8 mice each).

We examined whether output of TRPM8 induces behavior for warming the sole skin for regulation of skin temperature against cooling (Fig. 2). A free-moving mouse was placed in a cage with two floor temperatures for thermal preference test. When room temperature was neutral (27°C), stay time on 35°C floor was similar between wild-type and TRPM8-deficient mice, showing that TRPM8 is not involved in this behavior at 27°C. When room temperature was 10°C, stay time on 35°C floor was increased in both mice. However, stay time on 35°C floor in wild-type mice was longer than that in TRPM8-deficient mice. This indicates that TRPM8 comparators induce heat-seeking behavior for warming the sole skin against room cooling. This heat-seeking behavior corresponds to TRPM8-induced cold sensation [17], [21], [22], showing that TRPM8-induced cold sensation triggers the behavior for warming the sole skin. Cooling-induced TRPM8-independent behavior seems to be responses induced by non-TRPM8 receptors [28]. These results show that TRPM8 comparators work as thermostats of skin temperature, inducing behaviors for warming the sole skin against cooling.

### TRPM8-driven autonomic responses for warming the skin against cooling

#### TRPM8–induced activities of thermogenic BAT

**Figure 3:**
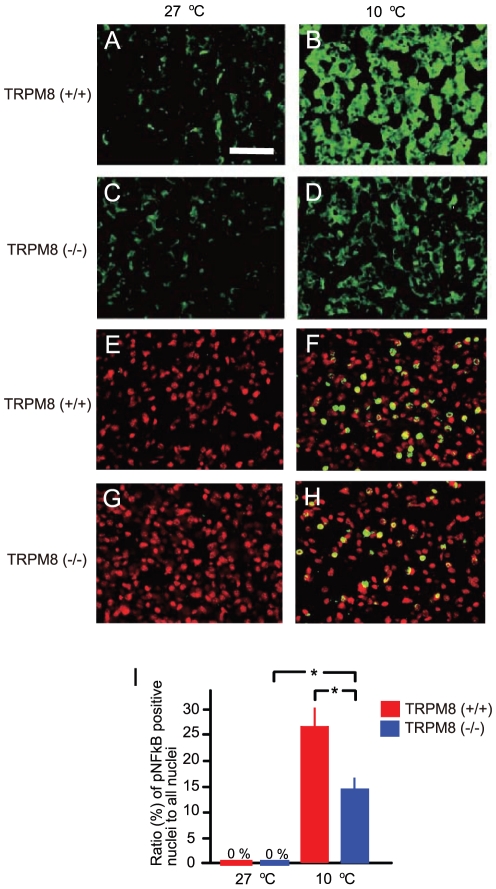
Wiring from TRPM8 to thermogenic BAT. (A–D) Fluorescence signal of UCP1 in BAT. At 27°C, UCP1 signal was low in both mice (A, C). When room temperature was 10°C, UCP1 signal was increased in both mice (B, D). However, UCP1 signal in wild-type mice (B) was largely enhanced, compared to that in TRPM8-deficient mice (D). Scale bar  = 50 µm. (E–H) Fluorescence signals of pNFkB in BAT nuclei. Cell nuclei were represented by red. At 27°C, fluorescent signals of pNFkB were not observed in both mice (E, G). At 10°C, the number of pNFkB positive nuclei (yellow) was increased in both mice (F, H). (I) Ratio of pNFkB-positive nuclei to all nuclei in BAT, counted from data as exemplified by E–H. Ratio was obtained in a field containing about 100 nuclei (n = 10 fields from 2 mice). The ratio of pNFkB-positive nuclei in wild-type mice was higher than that of TRPM8-deficint mice at 10°C.

Pathways from cooling-sensitive receptors to thermogenic brown adipose tissues (BAT) are well characterized [29], [30]. We examined whether TRPM8 channels induce BAT activities against room cooling (Fig. 3). To study cooling-induced BAT activation, mice were put at 10 or 27°C for 1h. When room temperature was 27°C, fluorescence of UCP1 was low in both TRPM8-deficient and wild-type mice (Fig. 3A, C). When room temperature was 10°C, UCP1 signals were increased in both mice (Fig. 3B, D), but the signal strength in wild-type mice was higher than that of TRPM8-deficient mice (Fig. 3B, D). To confirm this observation, we examined cooling-induced activities in phosphorylated NFkB (pNFkB) in nuclei, as a marker of BAT cell activities [23], [31]. At 27°C (Fig. 3E, G), fluorescence of pNFkB was detected in neither TRPM8-deficient nor wild-type mice. At 10°C, the number of pNFkB-positive nuclei was increased in both mice (Fig. 3F, H), but the ratio of the number of pNFkB-positive nuclei to all nuclei in wild-type mice was higher than that in TRPM8-deficient mice (Fig. 3I). These results show that TRPM8 induces activities of thermogenic BAT used for warming the skin in response to cooling.

#### Menthol- and cooling-induced TRPM8-driven thermogenic responses

**Figure 4:**
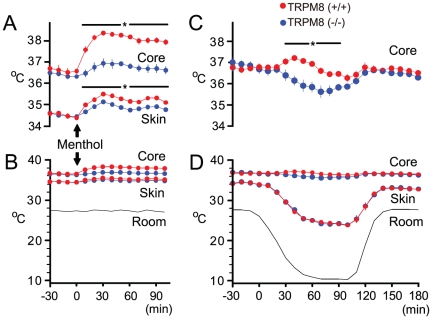
TRPM8-driven autonomic heat-gain responses. (A, B) Menthol-induced changes in core and skin temperatures, drawn in different temperature scales. When menthol was applied to the whole trunk skin (arrows) at 27°C (B), TRPM8-dependent rise in core temperature (1.3°C) and in skin temperature (0.4°C) occurred (n = 10 mice each). (C, D) Room cooling-induced changes in core and skin temperatures. Core temperatures were drawn in different temperature scales. When room temperature was decreased from 27 to 10°C (D), TRPM8-dependent small rise (1.0°C) occurred in core temperature (C, D), but skin temperatures were severely decreased from 34.2 to 24.1°C in both TRPM8-deficient and wild-type mice (n = 8 mice each).

We examined whether TRPM8 in the skin induces thermogenic responses for warming the skin. When room temperature was 27°C, core temperature was 36.8°C and skin temperature was 34.2°C in both mice (Fig. 4). This shows that TRPM8 is not involved in maintaining normal body temperatures at 27°C, consistent with the earlier study [17]. When menthol was applied to the whole trunk skin of mice at 27°C (Fig. 4A,B, arrows), TRPM8-dependent rise occurred in core (1.3°C) and skin temperatures (0.4°C).

In contrast, when room was cooled from 27 to 10°C (Fig. 4C, D), core temperature of TRPM8-deficient mice was decreased (Fig. 4C), but that of wild-type mice was increased slightly. The TRPM8-dependent difference (1.0°C) reflects TRPM8-induced thermogenesis in the core. By the same room cooling, however, skin temperature was decreased severely from 34.2°C to 24.1°C in both mice (Fig. 4D), as if regulation is not functioning. Room cooling-induced decrease in skin temperature in wild-type mice is similar to the earlier study of rabbits [32]. These results indicate that TRPM8-induced thermogenesis in the core is not powerful enough to warm the skin against cooling.

## Discussion

### TRPM8 is not transducer of temperature

It has generally been assumed that a sensory receptor is transducer that changes physical quantity into the firing-rate code of nerve impulses, and that sensation appears by decoding the code [7]. Along this line, a target neuron receiving impulses sent from thermo receptors has been assumed to be the “thermostat” that compares skin temperature with a set-point temperature at the code level [1], [3], [4], [8], [15].

However, the present study of TRPM8 shows that the above assumptions are not valid as a whole. TRPM8 is a polymodal receptor, responding to temperature, menthol, icilin and other chemicals [10], [14], [17], [27]. This is strong evidence to reject the view that TRPM8 is transducer of temperature, because TRPM8 does not respond to skin temperature solely. From the very beginning, the view that a mouse measures skin temperature with receptor is inadequate, for measurement is done exclusively by a person who knows the meaning of temperature (e.g., 28.4°C) and the principle of measurement. Thus, the hypothesis that a cerebral target neuron is the “thermostat” of skin temperature [1], [3], [4], [8] is not valid.

Temperature (e.g., 22.4°C) or menthol concentration (e.g., 100 µM) is physical quantity, and completely different from subjective feeling that the skin is cold. Therefore, the view that temperature or menthol concentration is transformed into cold sensation by coding-decoding system is not reasonable.

### TRPM8 is thermostat of skin temperature

**Figure 5:**
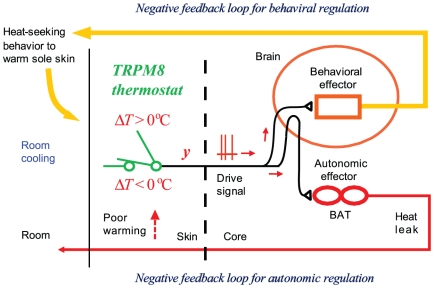
Schematic representation of the present findings. Cooling-sensitive TRPM8 is directional comparator of skin temperatures, inducing output (*y*) when Δ*T* ( =  *T* – 28.4°C) is negative (Fig. 1). If negative Δ*T*-dependent output (*y*) induces thermoregulatory responses to warm the skin recursively, negative feedback loops having 28.4°C as set-point temperature are formed and TRPM8 functions as thermostat for minimizing Δ*T* against cooling. In behavioral regulation, when room is cooled, TRPM8 induces behavior to move to heated floor to warm the sole skin, which effectively maintains temperature of the sole skin against cooling. In autonomic regulation, when room is cooled, TRPM8 induces a small rise in core temperature, but skin temperature is severely decreased, as if regulation of skin temperature is not functioning.

In contrast, the present study shows that TRPM8 is polymodal comparator of temperature, menthol concentration and icilin concentration. The present experiments show that output of TRPM8 induces responses to warm the skin, which indicates that TRPM8 acts as thermostat of the skin temperature against cooling (Fig. 5).

TRPM8-induced afferent impulses are drive signals, which do not convey a code for skin temperature to target neurons. Then, how is low skin temperature related to cold sensation? TRPM8-induced impulses should excite a cerebral target neuron involved in cold sensation, which triggers behavior to warm sole skin. Thus, we assume that information to induce cold sensation is innately stored in a target neuron. If not stored, cold sensation would not be elicited even when a target neuron is excited by arriving impulses. Electric stimulations of local areas of the somatosensory cortex in conscious persons induce feeling that various spots of the whole-body skin are touched [33]. This famous experiment supports the present assumption that information to induce cold sensation is stored in a target neuron. Cell theory states that a cell is the basic unit for structure and function of all plants and animals [34], which gives the basis for the modern cell biology [35]. This cell theory also supports the present assumption that information to induce cold sensation is stored in a target neuron, because cold sensation is a critical function to survive in low ambient temperatures. These explanations are also applicable to menthol- [36], [37] and icilin-induced cold sensation [38].

In autonomic regulation, when room is cooled deeply, the skin temperature is severely decreased in both mice (Figs. 4), as if regulation of the skin temperature is not functioning. This is an unexpected finding that TRPM8-induced autonomic responses are less powerful to maintain the skin temperature against cooling. To maintain skin temperature against cooling (Fig. 4), TRPM8-induced thermogenesis should warm the core drastically. However, an excessive rise in core temperature is harmful for mice. Thus, TRPM8-induced autonomic regulation of skin temperature has limited power in nature.

Many investigators have been interested in homeostasis of core temperature in mammals [1], [3], [4], [8]. When room temperature is decreased from 27 to 10°C (Fig. 4D), skin temperature is decreased from 34.2°C to 24.1°C but core temperature is maintained near 36.5°C in both mice. The difference between core temperature and room temperatures is as high as 26°C. Thus, there should be thermostat working for homeostasis of core temperature. Intraperitoneal injection of 5′-AMP induces torpor-like response, in which core temperature is reduced from normal level to ambient temperature [39]. This suggests that autonomic thermoregulatory system of core temperature is a device inhibited by 5′-AMP. However, the thermostat of core temperature remains unresolved.

## Materials and Methods

### TRPM8-deficient mice

TRPM8 (−/−) and TRPM8 (+/+) mice were obtained by breeding of TRPM8 (+/−) and TRPM8 (+/−) mice. Genotypes of mice were checked by allele specific PCR from tail genomic DNA [21]. TRPM8-deficient and wild-type mice were housed under standard conditions (27°C; 12 h light/dark cycle; lights on at 6:00 a.m.) with food and water *ad libitum*. Male mice (2–6 months old) were used for experiments. All protocols were approved by Animal Research Committee of Kyoto University (Approval ID: Graduate School of Informatics 19-4).

### Ratiometric calcium imaging

Doxycycline-inducible TRPM8-expressing HEK293 cells were established by Flp-In T-Rex system (Clontech, La jolla, CA), as described previously [40]. Briefly, TRPM8-expressing cells on a collagen-I-coated coverslip were loaded with 2 µM Fura-2 AM. After washing with Krebs' solution, these cells on a coverslip was placed on a recording chamber mounted on the stage of fluorescence microscope (BS50WI, Olympus). With a digital image analysis system (AQUACOSMOS; Hamamatsu Photonics, Hamamatsu), intracellular Ca^2+^ ion concentration was measured by Fura-2 fluorescence ratio (F_340_/F_380_, excitation 340/380 nm and emission 510 nm), and displayed as TRPM8 activity.

### Heat-seeking behavior

A plastic cage (16 cm ×27 cm) was placed in the incubator. Floor of a cage was divided into two zones. One half (16 cm ×13.5 cm) was kept at 35°C with a heating pad placed under the cage and the other half was unheated. A mouse was transferred to the center of a test cage. Behavior was recorded by a digital video camera (30 frames/s) for 30 min. Time on 35°C floor (%) was measured with Image J for *last 20 min.*

### Immunofluorescence

We used UCP1 and pNFkB as markers of BAT activation. UCP1 is a protein for heat production in BAT [41]. Localization of pNFkB is related to BAT activation [23], [31]. To study cooling-induced BAT activation, mice were put at 10 or 27°C for 1 h. After anesthesia with pentobarbital, BAT was harvested. BAT tissues were transferred into OCT compounds (Sakura, Tokyo, JAPAN), frozen, and sectioned (20 µm-thick) with a cryostat. Sections were fixed in 10% (v/v) formalin in 0.1 M phosphate-buffered saline (PBS) for 10 min. After washing with 0.1 M PBS, these sections were blocked for 30 min with 10% (v/v) normal donkey serum in 0.1 M PBS containing 0.2% Triton X-100. BAT sections were incubated with anti-UCP1 antibody (1∶1000, CHEMICON, Themecula, CA) or rabbit anti-pNFkB p65 (Ser 276) antibody (1∶100, Cell Signaling Technology). After washing, sections were incubated with AlexaFluor 488-conjugated anti-rabbit IgG (H+L) (1∶1000, Molecular Probes, Eugene, OR) for 2 h at room temperature. Cell nuclei in BAT were visualized with Hoechst 33342 (1∶50000, invitrogen, Eugene, OR). Fluorescent images of BAT sections were captured by fluorescence microscope (Nikon ECLIPSE E600).

### Temperature measurement and stimulation

Mice were implanted with E-mitter probe (minimitter, Bent, OR) in peritoneal cavity under pentobarbital anesthesia (70 mg/kg). 7 to 14 days after implantation, abdominal temperature was measured as core temperature. Mean temperature of the whole back skin was detected with thermography (Thermotracer TH3100MR, NEC San-ei Instrum, Kyoto, Japan) as skin temperature. By using anesthetized mice (n = 5), skin temperature of a thermography (15–33°C for room cooling experiment and 30–35°C for menthol application experiment) was calibrated with that of a thermocouple fixed on the back lumber with a tape.

Free-moving mice were put in an incubator (CRB-41, HITACHI; 62 cm ×50 cm ×132 cm) with a built-in regulator, and temperature in the incubator was used as room temperature. For thermal stimulation, room temperature was decreased from 27°C (neutral temperature) to 10°C. Room temperature was sampled every 10 min.

For menthol application, mice were anesthetized with 5% isoflurane for 30 sec. Menthol (*l*-menthol 10% w/v (640 mM), Wako, Japan) in ethanol was applied once to their whole trunk skin with paper (Kimwipes 6×5 cm) soaked with 600 µl solution of menthol.

### Statistical analysis

Data were represented as mean ± SEM. Comparisons of mean values between two groups were performed by one-tailed *t*-test. Statistical significance level, α, was set as 0.01 (*: p<0.01).
